# Translating evidence: pain treatment in newborns, infants, and toddlers during needle-related procedures

**DOI:** 10.1097/PR9.0000000000001064

**Published:** 2023-02-16

**Authors:** Denise Harrison, Mariana Bueno

**Affiliations:** a Department of Nursing, The University of Melbourne, Melbourne, Victoria, Australia; b School of Nursing, The University of Ottawa, Ottawa, ON, Canada; c Murdoch Children's Research Institute, Melbourne, Victoria, Australia; d Royal Children's Hospital, Melbourne, Victoria, Australia; e The Hospital for Sick Children, Toronto, ON, Canada

**Keywords:** Newborn, Infant, Pain, Implementation science, Sucrose, Breastfeeding, Distraction

## Abstract

**Introduction:**

Treatment of pain in preterm, sick, and healthy newborns and infants and toddlers (up to 2 years of age) is consistently reported to be inadequate, and effective strategies are poorly implemented.

**Objectives:**

To present existing evidence of effective pain treatment strategies during needle-related procedures and to highlight initiatives focused on translating evidence into practice.

**Methods:**

This Clinical Update focuses on the 2022 International Association for the Study of Pain Global Year for Translating Pain Knowledge to Practice in the specific population of newborns, infants, and toddlers. Best evidence is reviewed, and existing knowledge translation strategies and programs available to implement evidence into practice are presented.

**Results:**

Effective strategies for newborn and young infants during frequently occurring needle procedures include small volumes of sweet solutions, breastfeeding, or skin-to-skin care when feasible and culturally acceptable. In addition, strategies such as nonnutritive sucking, positioning, swaddling, gentle touch, facilitated tucking, and secure holding can be used. For toddlers, the evidence is less robust, and discerning between pain and distress is challenging. However, strategies recommended for needle-related procedures include upright secure comfort holding by parents/caregivers, age-appropriate distraction, and topical anesthetics. Translation of effective pain management needs to involve the family, who need to be supported and empowered to comfort their child during painful procedures. Organizational, nationwide, and global initiatives aimed at improving implementation of effective pain treatments exist.

**Conclusion:**

There is evidence of effective pain management strategies for newborns, infants, and toddlers, and a great deal of effort is being made to translate knowledge into action.

## Abstract 2

Evidence and knowledge translation tools are available for preventing and minimizing procedural pain in newborns, infants, and toddlers. Improving pain management practices for this population must be a priority.

**Key Points**

Carefully monitor need for bloodwork and other invasive procedures.
Effective and safe pain treatment strategies during procedures for sick and healthy newborns and infants include breastfeeding, skin-to-skin care, and sweet solutions.
For toddlers during needle procedures, consistent use of (1) upright secure comfort holding by parents/caregivers; (2) age-appropriate distraction; and (3) topical anesthetics are recommended. Sweet solutions may also help to reduce pain and distress in this age group.
Involving and empowering parents/caregivers during painful procedures is crucial.

## 1. Introduction

Sick and preterm newborns are exposed to repeated invasive skin breaking painful procedures throughout their hospitalization, with no or minimal use of known effective pain management strategies.^9,10,12,28,31,34^ Studies focusing on hospitalized children show similar information that hospitalized infants and children are exposed to frequent repeated painful procedures with inconsistent use of pain management strategies.^41,43,45^ Safe and effective evidence-based strategies exist to reduce pain and distress during these frequently occurring painful procedures. Heel lancing is by far the most commonly performed painful procedure in hospitalized newborns and young infants,^28^ whereas venipuncture, peripheral intravenous placement, and needle-related procedures classified as “needle pokes” are more commonly reported in hospitalized children.^43,45^ In all likelihood, these numbers of recorded painful procedures underestimate the true burden of pain because they rely on clinical documentation and rarely account for the pain intensity of the procedure, number of attempts, length of the procedure, and skill of the health care provider. Preventing pain is the best way to reduce burden of pain. Each and every decision for ordering blood sampling for diagnostics and monitoring of current status needs to be carefully made, based on health status, need for monitoring, current treatment, and treatment plans. Reducing the number of invasive procedures without compromising the health of hospitalized newborns, infants, and toddlers needs to be a priority in all settings.

The following section outlines evidence-based strategies, based on synthesized evidence from systematic reviews, for reducing pain during painful procedures in newborns, infants, and toddlers (Box 1). This will be followed by knowledge translation initiatives in place to promote widespread use of knowledge, including system-wide and national and global strategies. For information regarding pain management for surgery and underlying diseases, including opioid analgesics, see Clinical Update Pediatric pain treatment and prevention for hospitalized children.^14^ Note, “mother” is used and recognized in the context of breastfeeding and skin-to-skin care (SSC) as the birth parent throughout this update.

## 2. Pain management in neonates

There are 3 strategies that have been most extensively studied and shown to reduce pain in preterm and hospitalized term newborns during painful procedures. As summarized in Tables 1 and 2, these strategies are breastfeeding,^2,35^ SSC,^23^ and small volumes of sweet solutions (sucrose or glucose).^5,16,18,22,39^ In addition, rocking and holding, swaddling and containment such as facilitated tucking, and nonnutritive sucking can be considered as adjuvant treatments to use in addition, especially when using sweet solutions.^29^ Although less studied in the context of pain, the role of parents in providing developmental care interventions including gentle touch cannot be underestimated.^33^

**Table 1:** Systematic reviews breastfeeding and skin-to-skin care.

Author, y	Population	Painful procedure(s)	Intervention	No. of Trials	No. of Participants	Effective? yes/no
Breastfeeding or breast milk						
Shah et al., 2012^35^	Healthy term newborns	HL (16) VP (4)	Breastfeeding Breast milk	10 10	1075 996	Yes No
Benoit et al., 2017^2^	Newborns (term = 15, preterm = 6)	HL (10); IM (6); VP (3); ROP (1); tape removal (1)	Breastfeeding Breast milk	14 7	1517 675	Yes No
Harrison et al., 2016^19^	Infants 1–2 mo	IM (10)	Breastfeeding	10	1066	Yes
SSC						
Johnston et al., 2017^23^	Newborns (term = 7, preterm = 18)	HL (19); IM (4); VP & HL (1); VP (3); ROP (1); tape removal (1)		25	2001	Yes

HL, heel lance; IM, intramuscular injection; ROP, retinopathy of premature eye examination; SSC, skin-to-skin care; VP, venipuncture.

**Table 2:** Systematic reviews of sweet solutions.

Author, y	Population	Painful procedures(s)	Intervention	No. of trials (and infants)	Effective? yes/no
Tian (author's own team's work)	Newborns (term = 12, preterm = 15, both = 5)	VP (10); HL (7); ROP (6); AP (4); LP (1); PICC (1); all (1); repeated (2)	Sucrose and glucose	32 (4786)	Yes
Huang et al., 2019^22^	Newborns (term = 22, preterm = 9)	HL (15); VP (9); AP (4); LP (2); ROP (1)	Sucrose and glucose	31 (4999)	Yes
Harrison et al., 2017^18^	Newborns (cumulative meta-analysis)	Most frequent: HL (79); VP (24); ROP (11); IM (11)	All sweet solutions	168 (>1500)	Yes
Stevens et al., 2016^39^	Newborns (term = 38, preterm = 31, both = 5)	HL (38); VP (9); ROP (7); VP & HL (1); AP (1); IM or SC (6); circumcision (4); NGT (3); all (3); catheterization (1); Echo (1)	Sucrose	74 (7049)	Yes
Bueno et al., 2013^5^	Newborns (term = 24, preterm = 9, both 5)	HL (19); VP (10); IM or SC (4); ROP (1); VP & HL (1); HL & suction (1); PICC (1); circumcision (1)	Glucose (+1 each for artificial sweeteners, honey, mannitol)	38 (3785)	Yes
Gao et al., 2016^16^	Newborns (term = 2, preterm = 6)	All painful procedures (5); specified procedures (3)	Sucrose	8 (782)	Yes
Chen et al., 2016^8^	Infants 1–12 mo	All IM	Sucrose and glucose	20 (2376)	Yes
Harrison et al., 2010^20^	Infants 1–12 mo	All IM	Sucrose and glucose	14 (1618)	Yes
Kassab et al., 2012^26^	Infants 1–12 mo	All IM	Sucrose and glucose	14 (1551)	Yes
Harrison et al., 2015^21^	Children 1–16 y	IM or SC (6); VP (1); or VP or FP (1)	Sucrose (toddlers) and sweet chewing gum (children)	8 (808)	Insufficient evidence in toddlers; ineffective in children

AP, arterial puncture; Echo, echocardiogram; FP, finger prick; HL, heel lance; IM, intramuscular injection; NGT, nasogastric tube; PICC, peripheral inserted central catheter; ROP, retinopathy of premature eye examination; SC, subcutaneous injection; VP, venipuncture.

A discussion on the pain assessment methods used during these studies is beyond the scope of this clinical update focusing on translating effective pain treatment into action. However, pain outcomes reported are based on various well-accepted and psychometrically tested behavioral outcomes (crying duration, facial expressions of pain, and unidimensional composite pain scores), multidimensional composite pain scores, and less consistently, physiological indicators.^4^ However, it is important to acknowledge that there is no gold standard of pain measurement, and it remains challenging to differentiate pain from stress in all ages, especially critically ill neonates.^4^

### 2.1. Breastfeeding during painful procedures

#### 2.1.1. Overview and mechanism of action

Breastfeeding before and during short-lasting needle-related painful procedures, when possible, feasible, and culturally acceptable, reduces behavioral and physiological responses to pain. Mechanisms are considered multifactorial and include tactile stimulation through the SSC, smell, heartbeat, and chest movement of the mother, as well as sucking, the slightly sweet taste of breast milk, and possibly, endogenous opiates present in the breast milk.^2,35^

#### 2.1.2. Systematic review findings

The most recent Cochrane systematic review of breastfeeding for procedural pain reduction is now a decade old.^35^ This review included 20 trials in total: 16 during heel lancing and 4 during venipuncture. All included healthy term or near-term newborns. Ten studies focused on breastfeeding and 10 focused on expressed breast milk. Breastfeeding was shown to reduce pain based on crying duration, facial expression scores, composite pain scores, and heart rate changes from baseline. For example, duration of crying was able to be pooled for 5 studies, with results showing a mean difference (MD) of −41 seconds (95% confidence interval [CI], −50 to −33) in the breastfeeding group compared with no intervention. Similarly, the well-established multidimensional Premature Infant Pain Profile (PIPP) scores were pooled for 3 studies and showed significantly lower scores in the breastfeeding group compared with a group administered water (MD −6; 95% CI, −7 to −4) and compared with being held swaddled by mothers (MD −7; 95% CI, −9 to −6). However, the same analgesic effects were not seen with supplemental breast milk. As breast milk contains around only 7% lactose, which is the least sweet of the sugars (fructose > sucrose > glucose > lactose),^3^ the mild sweet taste most likely contributes little to analgesia in isolation (eg, delivered by oral syringe or via pacifier).

More recently, Benoit et al.^2^ published an updated systematic review and included 21 additional trials published from 2011 to 2016. Most procedures studied were heel lance (10), followed by intramuscular injection (6), of which 3 included infants beyond the newborn period. Three studies focused on pain during venipuncture and one each on eye examination and tape removal. Breastfeeding was the intervention in 14 studies and supplemental breast milk in 6 studies, and in 1 study, breastfeeding was compared with breast milk and sucrose. Findings were the same as above, with all studies comparing breastfeeding to other interventions reporting consistent analgesic effects during painful procedures but not for breast milk.

A commonly asked question concerns whether formula feeding similarly reduces pain. Only 1 study was included in the Cochrane systematic review of breastfeeding or breast milk for newborns,^35^ which evaluated formula feeding compared with breastfeeding, no treatment, glucose, nonnutritive sucking, and holding.^44^ This single study showed that formula feeding was equally as effective as breastfeeding. No such study was included in the more recently published systematic review.^2^

#### 2.1.3. Bottom line

If newborns are able to breastfeed, and breastfeeding is considered possible, feasible, and culturally acceptable during needle-related painful procedures such as heel lance, intramuscular or subcutaneous injections, venipuncture for blood sampling or venous line placement, breastfeeding should be considered as the first-line intervention. For this to occur, health care providers need to facilitate mothers to be present, comfortably seated, and able to be involved at the time of procedures and support them to breastfeed their newborn during procedures. As all studies to date have included healthy medically stable newborns, it is not known if breastfeeding in sick newborns is feasible and effective. Further studies are warranted in this area.

### 2.2. Skin-to-skin care during painful procedures

#### 2.2.1. Overview and mechanism of action

Holding newborn infants SSC, with the infant wearing only a diaper, against a parents' chest before and during short-lasting, needle-related painful procedures reduces behavioral and physiological responses to pain. Mechanisms are considered multifactorial and include tactile stimulation through the skin-to-skin contact, warmth, smell, heartbeat and movement of the mother, father, or other caregiver. Nearly all studies have focused on mothers.^23^

#### 2.2.2. Systematic review findings

A Cochrane systematic review of SSC for procedural pain reduction was published in 2017.^23^ This systematic review included 25 studies with 2001 newborns; 18 studies include preterm and 7 included term newborns (Table 1). Most studies (19, 76%) included focused on heel lance and included 1065 newborns. Four studies focused on intramuscular injection, one included both venipuncture and heel lance and one on tape removal. All except 2 studies focused on mothers only: 1 study compared mothers with fathers and 1 study compared mothers with an alternate female. Overall, results showed that SSC significantly reduced pain based on behavioral responses, heart rate, and composite pain scores. For example, PIPP scores during heel lance, at 30 seconds into the procedure, were pooled in a meta-analysis and showed a significant reduction for SSC compared with no treatment (MD −3.21, 95% CI, −3.94 to −2.47), and heart rate during heel lance was statistically significantly lower (−11 beats/minute; 95% CI, −14 to −8) in the SSC group.

#### 2.2.3. Bottom line

If preterm, sick, and healthy newborns are able to be held SSC during needle-related painful procedures such as heel lance, intramuscular or subcutaneous injections, venipuncture for blood sampling or venous line placement, SSC should be facilitated. For this to occur, health care providers need to facilitate mothers or other caregivers to be present, comfortably seated, and able to be involved at the time of procedures and support them to hold the infants SSC during procedures.

### 2.3. Sweet solutions during painful procedures

#### 2.3.1. Overview and Mechanism of Action

The administration of small volumes of sweet solutions, specifically, sucrose and glucose, onto the infant's tongue 1 to 2 minutes before single, short-lasting painful procedures reduces pain during and after procedures. Sweet taste analgesia is thought to have 2 mechanisms: (1) immediate calming because of the strong (sweet) taste and (2) ongoing calming because of a sweet taste–mediated endogenous opioid release.^39^

#### 2.3.2. Systematic review findings

Sweet solutions are the most extensively investigated intervention for procedural pain relief in newborns. A number of systematic reviews have been published over the past 20 years confirming the effectiveness and safety of sucrose and glucose during different painful procedures in diverse populations of newborns and infants beyond the neonatal period^8,18,20,22,26,39^ (Table 2). In the largest of these systematic reviews, a cumulative meta-analysis (CMA) involving all types of sweet solutions for newborns included 168 studies involving different painful procedures. Results demonstrated that sweet solutions reduced behavioral indicators of pain since the earliest studies published.^18^ For example, CMA for crying time included 29 trials and 1175 neonates and showed a reduction in crying time of 23 seconds in favor of sweet solutions (95% CI, −29 to −18). When pain scores were standardized to a score out of 10, and pooled for meta-analysis, 50 trials with 3341 neonates were included. Results showed a statistically significant reduction of 0.9 (95% CI, −1.1 to −0.7).

In addition, Gao et al.^16^ investigated the safety and efficacy of repeated administration of sucrose in a systematic review of 8 studies. Conclusions were made that sucrose continued to reduce pain during repeated episodes of painful procedures. Although no adverse effects of sucrose administration were reported, a subanalysis of 1 trial of repeated doses of sucrose during the first week of life reported that preterm infants <31 weeks' gestational age who received >10 doses of sucrose daily had lower neurobehavioral scores at 36 weeks' gestational age compared with infants who received fewer sucrose doses.^24^ These differences, however, had normalized by 40 weeks' corrected gestational age. Stevens et al.,^37^ in a larger study in which infants received sucrose or water for all procedures in the first month of life, found no differences in neurobehavioral outcomes. More recently, Campbell-Yeo et al.^7^ evaluated sustained safety and efficacy of repeated doses of sucrose alone for all painful procedures over the course of a hospitalization compared with SSC alone and the combination of the 2 strategies. No differences in pain outcomes or neurobehavioral outcomes at 32 and 36 weeks' corrected gestational age were reported between the groups.

An important question over the years has focused on how much sucrose or glucose to use. Findings of the systematic reviews highlighted variability in sucrose and glucose concentrations and volumes used. However, in a multisite trial including 245 neonates, which evaluated 3 different volumes of 24% sucrose, the smallest volume of 0.1 mL was found to be just as effective as larger volumes during heel lancing.^38^

#### 2.3.3. Bottom line

Small volumes (0.1 mL) of sweet solutions (sucrose and glucose of at least 15%–20% concentration) are effective and safe for preterm and term infants during short-lasting frequently performed needle-related painful procedures. Only small volumes are required for analgesic effects (2–3 drops), administered on the top of the tongue, around 1 to 2 minutes before the procedure, just before the procedure, and repeated throughout the procedure if the infant shows signs of pain. The combination of sweet solution with nonnutritive sucking (NNS), SSC, facilitated tucking, and swaddling is recommended.

## 3. Pain management in infants and toddlers

The evidence for analgesic effects of pain management interventions during needle-related painful procedures for infants beyond the newborn period, and up to 2 years of age, is less robust than that for newborn infants. In addition, for toddlers, it is challenging, and often not possible, to discriminate pain from distress. Toddlers may demonstrate similar levels of distress to painful and nonpainful procedures or already be crying before a needle insertion, making accurate assessment of potentially effective pain management interventions difficult.^11^ The reasons for such distress are multifactorial and relate to fears associated with unfamiliar environments and unfamiliar people, parental separation, and being positioned lying down during procedures. For example, in a trial of sucrose in children aged 12 to 36 months, most of the children were already crying at baseline, before the insertion of the cannula.^30^

Regardless, there is evidence of strategies that have been shown to reduce pain in infants beyond the newborn period. In addition, there are strategies with evidence of analgesic effects in older children, which may also be assumed to reduce pain in younger children. In the following section, evidence for the following strategies will be summarized: sweet solutions, breastfeeding or formula feeding, topical anesthetics, upright secure holding, and distraction. As per newborn pain management, supporting parents/caregivers to be involved during painful procedures is essential.^17^

### 3.1. Sucrose during painful procedures

As shown in Table 2, the most recent systematic review of sweet solutions (sucrose and glucose) for infants beyond the newborn period up to 1 year of age focused only on vaccination pain.^22^ Findings highlighted that, as long as solutions were sufficiently sweet, small volumes of sucrose or glucose reduced crying duration during vaccination, by 21 seconds, and in the 3 minutes after vaccination, by 14 seconds. Sucrose or glucose can therefore be recommended for this age group. For maximum analgesic effects, however, repeating the dose is recommended shortly or just before commencing the procedure to ensure sustained analgesic effects.

For children older than 12 months of age, a systematic review of 8 trials published up to June 2014 and including 808 children found that the evidence was inconclusive for toddlers, and there was no evidence of efficacy for children older than 4 years of age. Since the publication of the systematic review, further studies have focused on toddlers.^13,25,30^ For example, 2 studies of sucrose during vaccination in children aged 15 months^13^ and 10 to 18 months^25^ reported statistically significant reductions in crying duration. Mean crying duration in the sucrose group was 18 seconds compared with 33 seconds in the control group in 15-month children^13^ and 52 seconds (SD 23) compared with 65 seconds (SD 19) in the control group in 18-month children.^25^

In addition, Modanloo et al.^30^ evaluated sucrose compared with water in 95 hospitalized toddlers during venipuncture, with topical anesthetic as the control condition. Sixty-one toddlers (72%) were aged 12 to 24 months and 24 (28%) were aged 24 to 36 months. Overall, sucrose did not statistically significantly reduce crying time or pain scores, and most infants were crying at baseline, before insertion of the needle.

### 3.2. Breastfeeding during painful procedures

As shown in Table 1, a systematic review of breastfeeding for needle pain beyond the newborn period, up to 1 year of age, included 10 studies with 1066 infants. All 10 studies focused on vaccination pain.^19^ Pooled data showed that breastfeeding clinically and statistically significantly reduced duration of crying time by 38 seconds and standardized mean pain scores by 1.73/10 during the vaccination procedure.^19^ Breastfeeding infants during vaccination can therefore be recommended. However, only 2 studies included infants older than 6 months; therefore, the evidence for efficacy, and also availability and feasibility of breastfeeding older infants and toddlers, is less well known. No studies in the systematic review compared bottle feeding with either formula or expressed breast milk. More recently, breastfeeding and bottle feeding with formula were compared in a study of vaccination pain in infants.^42^ Similar reductions in crying duration and pain scores were reported for both breastfeeding and bottle feeding compared with no treatment, highlighting that pain reduction with bottle feeding may also be because of the close contact between the caregiver and the infant, despite not being attached to the breast. Bottle feeding can therefore be recommended for when breastfeeding is not able to occur. To date, no studies outside the vaccination context have been identified. The efficacy, effectiveness, feasibility, and acceptability of breastfeeding older infants and toddlers during painful procedures other than vaccination is therefore not known. However, when possible, feasible, and practical, breastfeeding or bottle feeding could be considered for nonurgent needle procedures, depending on feeding practices and parent preferences, up to around 12 months of age.

### 3.3. Physical and psychological strategies during painful procedures

A systematic review of nonpharmacological management of infants' and young children's procedural pain included strategies with some, but limited, evidence of effectiveness for older infants and young children.^32^ Strategies with evidence of effectiveness included nonnutritive sucking (2 studies), touch or massage (2 studies), distraction with videos (2 studies), and structured nonparent distraction (1 study). However, both massage and nonparent distraction were no more efficacious than oral sucrose. Distraction by parents (6 studies) and distraction with toys (4 studies) were not shown to be effective. Most studies were considered low- to very low-quality evidence. Although upright holding was not specifically considered in this review, when possible and feasible, infants and toddlers should be securely held in an upright position to reduce distress by enhancing children's sense of control.^14,40^

### 3.4. Topical anesthetics

A systematic review of pharmacological strategies for vaccination pain included 15 studies of topical anesthetics in children <12 years of age.^36^ A meta-analysis including 13 studies and 1424 infants showed a mean reduction of standardized acute distress scores of 0.91 (95% CI, −1.36 to 0.47) with topical anesthetics when administered before vaccination.^36^ Topical anesthetics were therefore recommended during vaccination for infants and young children, and topical anesthetics during needle procedures are included in national and international recommendations for reducing pain during needle procedures.^40^ Depending on the brand used, the agents need to be left on the skin for 30 to 60 minutes for maximum analgesic effects. Table 3 includes a summary of evidence-based recommendations of pain management strategies.

**Table 3:** Summary of recommendations for needle pain reduction.

Strategy	Population	Details
Breastfeeding	Medically stable newborns and infants up to 12 mo of age and beyond if possible, feasible	When possible, feasible, and culturally acceptable, begin ∼5 min or so before procedure to ensure infant is attached and sucking. Continue throughout procedure. Note: Bottle feeding can be used if breastfeeding is not possible. If infant comes off breast or stops sucking bottle, allow them time to re-attach and begin sucking.
SSC	Preterm and term newborns	Commence around 10–15 min before procedure to ensure infant and parent/other caregiver are comfortable and settled. Continue throughout procedure.
Sucrose or glucose	Preterm and term newborns and infants up to ∼18 mo of age	Small volumes (∼0.1 mL) of at least 15% concentration. Give in aliquots ∼1–2 min before procedure and upon needle insertion, and repeat as required if infant cries. Use with pacifier if this is a normal part of infant's care. Use in addition to SSC as required and add physical strategies (swaddling, rocking, or holding) as appropriate.
Comfort position, facilitated tucking, and physical strategies	All ages	In addition to sucrose or glucose, and topical anesthetics if appropriate, use swaddling, facilitated tucking, rocking, holding, singing, upright secure holding, and NNS as required.
Topical anesthetics	All ages	Evidence less robust for newborns and ineffective for heel lance procedures. Recommended for use in combination with sweet solutions and other comforting strategies for more prolonged procedures including PICC line insertion. For older infants and toddlers, for elective needle procedures, apply 30–60 min before procedure.

NNS, nonnutritive sucking; PICC, peripheral inserted central catheter; SSC, skin-to-skin care.

### 3.5. Partnering with parents during painful procedures

Involving and empowering parents during painful procedures is essential to minimizing pain and distress. As per the previous section, breastfeeding, SSC, secure comfort positioning, and simply being present require parents to be involved, informed, and empowered and play a fundamental role in recognizing their child's pain.^15^ Parents report wishing to learn about pain and being involved during painful procedures.^6,27^ Implementing organized approaches such as family-integrated care, with a focus on pain management, may facilitate parents consistently taking more active roles with their health care providers.^1^

## 4. Implementing the evidence

Shifting knowledge into consistent action across diverse settings where newborns and young children undergo painful procedures requires producing knowledge into “usable” evidence, in the form of knowledge translation products, and coordinated efforts at local, national, and international levels. Usable evidence includes child, parent, and clinician-targeted knowledge translation products, such as videos, booklets, and position statements. Examples of child, parent/caregiver, clinician, and organizational-targeted knowledge translation products and initiatives are summarized in Table 4. These include a series of parent-targeted videos and written information, targeted at parents of newborns and young infants. In addition online web sites with embedded videos, patient and clinician stories, and recommendations on working collaboratively to reduce pain and stress during painful procedures.

**Table 4:** Usable Evidence and organizational initiatives.

Initiative, target audience, and URL	Content	Details
Be Sweet to Babies: Parents of newborns https://www.youtube.com/watch?v=L43y0H6XEH4&feature=youtu.be	Breastfeeding, SSC, and sweet solutions during heel lance and venipuncture	Brief (4 min) video posted on YouTube, 2016 Produced in 10 languages: English, French, Spanish, Arabic, Inuktitut, Farsi, Mandarin, German, Hindi, and Swiss Italian
Be Sweet to Babies: Parents of infants undergoing vaccination https://www.cheo.on.ca/en/clinics-services-programs/be-sweet-to-babies.aspx	Breast feeding and sucrose for pain management during vaccination	Three videos showing pain management during vaccination; includes breastfeeding a 2-mo infant and an 8-mo infant and sucrose for a 4-mo infant
Be Sweet to Moms and Babies: Clinicians caring for newborns undergoing blood sampling https://www.youtube.com/watch?v=lpZNwP7bnkg&feature=youtu.be	Nurse performing heel lance while healthy newborns are breastfeeding and being held SSC	Brief (<4 min) video, posted on YouTube 2019; focus is on clinician teaching and how to perform heel lancing while newborns are breastfeeding and held SSC
Power of a Parents' Touch: Parents of newborns https://m.youtube.com/watch?v=3nqN9c3FWn8&feature=youtu.be	SSC & breastfeeding during painful procedures	Brief (<3 min) video posted on YouTube, 2014; includes information about burden of pain, risk of adverse outcomes of poorly treated pain, and benefits of SSC & breastfeeding during painful procedures
Comfy booklet: Parents of newborns https://familynursing.ucsf.edu/sites/familynursing.ucsf.edu/files/wysiwyg/Comfy%20PDF%20ENGLISH%20Dec%2017.pdf	Parents' role in NICU and strategies to use in reducing stress and in comforting sick newborns	40-page multimedia booklet (2013) with information for parents, and embedded brief (<40 s) video clips
Children's Comfort-promise (Children's Hospital Minnesota): Parents/caregivers of hospitalized infants and children https://www.childrensmn.org/services/care-specialties-departments/pain-program/childrens-comfort-promise/	Hospitalized children's needle pain and parent education and engagement	Web page including written information and videos focusing on an organizational promise to minimize needle pain using 4 strategies: (1) topical anaesthetics; (2) sucrose or breastfeeding for infants 0 to 12 mo; (3) comfort positioning (including swaddling, SSC, or facilitated tucking for infants; sitting upright for children); (4) age-appropriate distraction
Help eliminate pain in kids & adults (HELP): Parents/caregivers of infants and children AND clinicians http://phm.utoronto.ca/helpinkids/resources1.html	Vaccination pain	Knowledge translation products showcasing evidence-based pain management during vaccination; involves partnership with universities, hospitals, and government and nongovernment organizations across Canada
Solutions for kids in pain (SKIP): Children, parents/caregivers of infants and children AND clinicians https://kidsinpain.ca/	Raising public awareness of child pain	Brings together pediatric pain specialists, knowledge users, consumers, and patient/caregiver partners across various sectors (eg, charities, governments, businesses, and health care); goals are to increase awareness of children's pain in Canada
ChildKind International https://childkindinternational.org/	Accreditation of hospitals caring for children	Recognition of institutional commitment to incorporate child pain management as a value

NICU, Neonatal Intensive Care Unit; SSC, skin-to-skin care.

## 5. Conclusion

Hospitalized newborns, infants, and young children require large numbers of painful needle-related procedures. There is robust evidence of analgesic effects of breastfeeding, SSC, and small volumes of sweet solutions for newborns. Although less robust, sufficient evidence exists of breastfeeding, sweet solutions, topical anesthetics, secure comfort positioning, and distraction for infants beyond the newborn period. For toddlers, it is difficult to discriminate between pain and distress; however, topical anesthetics, secure upright comfort positioning, and distraction during needle procedures are recommended. For all ages, supporting and empowering parents and caregivers to be present and involved is essential. Coordinated organizational, national, and global knowledge translation initiatives targeted at parents and clinicians aim to move knowledge into consistent use in diverse settings where infants and young children require painful procedures. Evidence and knowledge translation tools are available for preventing and minimizing procedural pain in newborns, infants, and toddlers. Improving pain management practices for this population must be a priority (Box 1).

Box 1. **Reducing needle pain.**

Offer/facilitate use of most appropriate strategy, depending on parent preference, infant/child's medical status, availability of the mother/alternate caregiver of all infants and children for all needle procedures.

(1). Newborns: Breastfeed or hold SSC before and during procedures or give small volumes (0.1 mL) of sucrose/glucose 1 to 2 minutes before procedure, with NNS if part of infants' normal care. Adjuvant interventions may include swaddling, facilitated tucking, rocking, or singing.
(2). One to twelve months: Apply topical anesthetics 30 to 60 minutes before procedure. Breastfeed before and during procedures or give small volumes (0.1 mL) of sucrose/glucose ∼1 minute before procedure, with NNS if part of infants' normal care. Repeat sucrose dose if required. Adjuvant interventions may include swaddling, rocking, singing, secure upright holding, or age-appropriate distraction.
(3). One to two years: Apply topical anesthetics 30 to 60 minutes before procedure. Use age-appropriate distraction and secure upright holding. For comfort positioning, “do not hold children down.” If able to, and preferred by mother, breastfeed before and during procedures. Small volumes of sucrose/glucose ∼1 minute and immediately before procedure, with NNS if part of child's normal care, may further assist to reduce pain.

## Disclosures

The authors have no conflicts of interest to declare.
